# EMERGING ADULT CAREGIVERS: PERCEPTIONS OF OLDER ADULT CARE RECIPIENTS, QUALITY OF CONTACT, AND AGEISM

**DOI:** 10.1093/geroni/igz038.3455

**Published:** 2019-11-08

**Authors:** Anastasia E Canell, Grace Caskie

**Affiliations:** Lehigh University, Bethlehem, Pennsylvania, United States

## Abstract

Approximately 12-18% of family caregivers to older adults in the U.S. are 18-25 years old (i.e., emerging adulthood), yet minimal research has focused on this subgroup of caregivers (Levine, 2005; Smyth, Blaxland, & Cass, 2011). Individuals’ perceptions of an older adult’s social role relate to their attitudes toward older adults as a group (Hummert, 1999; Kite & Wagner, 2002). However, whether perceptions that emerging adult caregivers hold of older adults are specific to the social role of “care-recipient” has not been studied. A sample of 210 informal caregivers (ages 18-25) were surveyed to collect qualitative responses regarding perceptions of an older adult care-recipient (age 65+) and to assess quality of contact with the care-recipient and ageist attitudes. Participants were asked to provide five adjectives describing their older adult care-recipient. Approximately 43% provided a set of adjectives in which 80%-100% were coded as positive adjectives (e.g., “active”, “wise”); similarly, half of the sample’s adjective sets contained 0%-25% negative adjectives (e.g., “helpless”, “obnoxious”). The quality of contact with the care-recipient was significantly correlated (p<.001) with the percentage of positive (r=.47) and negative (r=-.49) adjectives. Scores on the Fraboni Scale of Ageism were also significantly correlated (p<.01) with the percentage of positive (r=-.19) and negative (r=.20) adjectives. Overall, these emerging adult caregivers had generally positive perceptions of their older adult care-recipients, and these perceptions reflected the positive quality of contact with the care-recipient. Less ageist attitudes’ relationship with more positive and less negative perceptions may have implications for experiences within a caregiving dyad.

